# Multiplexing Stimulus Information through Rate and Temporal Codes in Primate Somatosensory Cortex

**DOI:** 10.1371/journal.pbio.1001558

**Published:** 2013-05-07

**Authors:** Michael A. Harvey, Hannes P. Saal, John F. Dammann, Sliman J. Bensmaia

**Affiliations:** Department of Organismal Biology and Anatomy, University of Chicago, Chicago, Illinois, United States of America; McGill University, Canada

## Abstract

Our ability to perceive and discriminate textures relies on the transduction and processing of complex, high-frequency vibrations elicited in the fingertip as it is scanned across a surface. How naturalistic vibrations, and by extension texture, are encoded in the responses of neurons in primary somatosensory cortex (S1) is unknown. Combining single unit recordings in awake macaques and perceptual judgments obtained from human subjects, we show that vibratory amplitude is encoded in the strength of the response evoked in S1 neurons. In contrast, the frequency composition of the vibrations, up to 800 Hz, is not encoded in neuronal firing rates, but rather in the phase-locked responses of a subpopulation of neurons. Moreover, analysis of perceptual judgments suggests that spike timing not only conveys stimulus information but also shapes tactile perception. We conclude that information about the amplitude and frequency of natural vibrations is multiplexed at different time scales in S1, and encoded in the rate and temporal patterning of the response, respectively.

## Abstract 2

In somatosensory cortex, stimulus amplitude is represented at a relatively coarse temporal resolution, while stimulus frequency is represented by precisely timed action potentials.

## Author Summary

When we slide our fingertip across a textured surface, small, complex, and high-frequency vibrations are elicited in the skin and our nervous system extracts information about texture from these vibrations. In this study, we investigate how texture-like vibrations are processed in primary somatosensory cortex (S1). First, we show that the time-varying amplitude of skin vibrations is encoded in the time-varying response rates of a subpopulation of S1 neurons. Second, we show that this same subpopulation of S1 neurons produces responses whose timing closely matches that of the vibrations: The frequency composition of the spiking patterns matches that of the stimulus, even for complex vibrations. We demonstrate that this temporal precision is behaviorally relevant by showing that the tactile perception of vibration is better predicted from neuronal responses when spike timing is taken into consideration than when it is not. The activity of S1 neurons is thus multiplexed at different time scales: Stimulus amplitude, which changes relatively slowly, is represented at a relatively coarse temporal resolution, while stimulus frequency is represented by precisely timed action potentials.

## Introduction

When we scan our finger across a textured surface, complex, high-frequency vibrations are elicited in the skin, and our ability to acquire information about surface microgeometry relies on the transduction and processing of these vibrations [1]–[4]. At the somatosensory periphery, the intensity of both simple and complex vibrations is encoded in the strength of the response these elicit in populations of mechanoreceptive afferents [5],[6]. The frequency content of skin vibrations, on the other hand, is conveyed through the timing of the response with millisecond precision [7], as illustrated by the well-documented phase-locking of peripheral fibers to sinusoidal stimuli [8],[9]. In primary somatosensory cortex (S1), neurons exhibit phase-locked responses to low-frequency sinusoidal stimuli (<100 Hz) [10], but the extent to which this temporal patterning is perceptually relevant is unclear [10]–[12]. Importantly, phase locking in S1 responses has been reported to disappear at higher frequencies [10], so the cortical mechanisms that mediate our ability to distinguish the spectral content of high-frequency vibrations remain to be elucidated. Finally, virtually nothing is known about how naturalistic (spectrally complex) vibrations are represented in cortex.

To investigate how information about vibratory amplitude and frequency is encoded in S1, we recorded single unit responses in areas 3b, 1, and 2 of awake Rhesus macaques (*Macaca mulatta*) to sinusoidal, diharmonic, and band-pass noise vibrations delivered to the glabrous skin of the hand. Stimuli comprised frequency components between 50 and 800 Hz and spanned the range of behaviorally relevant intensities. First, we found that the firing rate evoked in populations of cortical neurons encodes the instantaneous amplitude of skin vibrations. Second, we discovered a population of neurons in area 3b that exhibits phase locking in their responses to both simple and complex stimuli up to 800 Hz. This phase locking gradually disappears as one ascends the processing hierarchy to areas 1 then 2. Third, we confirmed using an information-theoretic analysis that stimulus amplitude and spectral content are represented using a multiplexed code, where information about amplitude is mainly conveyed in the firing rates of neurons on slow time-scales, while information about frequency content is carried by spiking patterns on fast time-scales. Finally, we show that the temporal patterning in the response of S1 neurons to vibrations shapes how they are perceived.

## Results

We recorded the responses of four Pacinian (PC) afferents and 211 well-isolated S1 neurons (area 3b: *n* = 69; area 1: *n* = 103; area 2: *n* = 39) to sinusoidal, diharmonic, and band-pass noise stimuli, varying over a broad range of frequencies and amplitudes.

### Cortical Representation of Stimulus Amplitude

**Figure 1:**
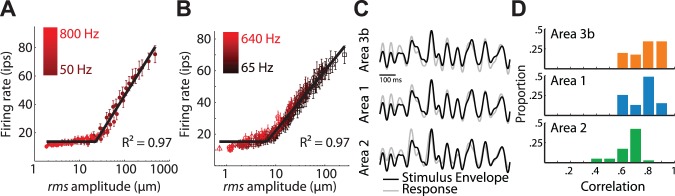
Stimulus amplitude versus firing rates. (A) Mean rate-intensity function for neurons in area 3b, computed from responses to sinusoids; marker colors change from dark to bright red with increasing stimulation frequency; error bars denote standard error of the mean. The shallow and frequency-independent increase in firing rate at low amplitudes is due to progressive neuronal recruitment and is not observed in the responses of individual neurons (see Figure S1A). (B) Rate-intensity function for neurons in area 3b, computed from responses to band-pass noise stimuli; colors change from dark to bright red as the center frequency of the noise increases; markers denote different noise conditions (triangle, noise_A_; circle, noise_V_; square, noise_P_, see Materials and Methods). For both individual neurons and neural populations, responses are independent of the frequency composition of the stimulus. (C) Hilbert transform of a sample noise stimulus (black) and smoothed time-varying population firing rates (gray) in areas 3b (top), 1 (middle), and 2 (bottom). The neural response is time-shifted to account for neural delays. (D) Mean correlation between the time-varying population response and the logarithm of the stimulus envelope in the three areas.

First, we wished to determine how the amplitude of vibrations was encoded in the responses of cortical neurons. We found that firing rates increased logarithmically with amplitude over the range tested for all three stimulus sets (Figures 1A, 1B, and S1), as is the case at the somatosensory periphery [5]. The slope of the rate-intensity function was steepest for area 3b neurons, shallowest for area 2 neurons, and intermediate for area 1 neurons (50, 30, and 10 spikes/log (µm) for areas 3b, 1, and 2, respectively). To investigate how the instantaneous amplitude of non-stationary stimuli was encoded in S1, we characterized the stimulus envelope using the Hilbert transform and assessed the extent to which variations in stimulus amplitude were reflected in variations in firing rates. We found that the time-varying firing rates were highly correlated with the logarithm of the stimulus envelope. In other words, the instantaneous stimulus amplitude was faithfully encoded in the instantaneous firing rates (Figures 1C, 1D, and S2). The correlation between instantaneous stimulus amplitude and firing rate was strongest in area 3b (mean ± standard error of the mean (SEM) = 0.78±0.01), and area 1 (0.75±0.02), and substantially weaker in area 2 (0.6±0.02), and these differences were significant (one way ANOVA, *F(2,141)* = 43.0, *p*<0.001).

### Cortical Representation of Frequency Content

The strength of the population response is almost completely independent of stimulus frequency (Figures 1A, 1B, and S3), as has been previously found for frequencies above around 50 Hz [10]. We found that only 3% of neurons exhibited significantly frequency-dependent responses to sinusoids, and ∼1% exhibited frequency dependent responses to noise (δR^2^ F-test, *p*<0.01); in these small populations of frequency-sensitive neurons, significant modulation was eliminated when frequency components below 100 Hz were excluded. The frequency independence of S1 responses to stimuli at frequencies above 100 Hz stands in contrast to the strong frequency dependence observed in the responses to stimuli in the flutter range (<100 Hz) [10],[12]. In conclusion, frequency information about high-frequency skin vibrations is not encoded in the firing rates of S1 neurons. In light of this, we next explored the possibility that the frequency composition of stimuli was encoded in the timing of cortical responses.

**Figure 2:**
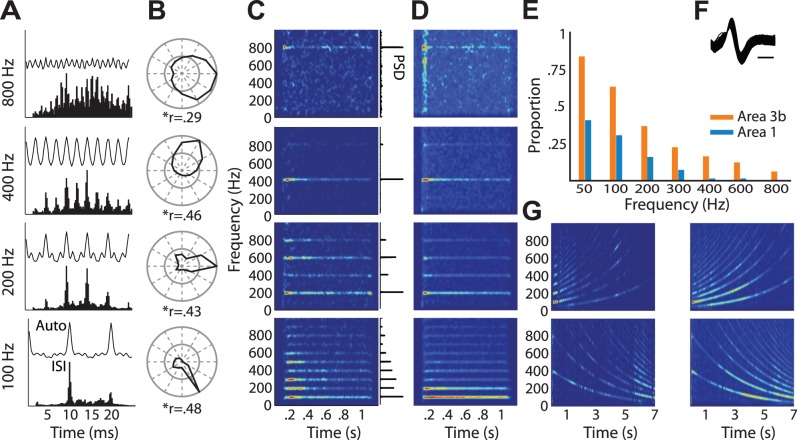
Temporal patterning in cortical responses to sinusoidal stimulation. (A) Autocorrelations (top) and interspike interval histograms (bottom) for one neuron in area 3b, whose responses are entrained with the stimulus up to 800 Hz. (B) Phase histograms along with vector strength (r) for the same neuron as in (A) showing action potentials tended to occur during a restricted phase of each stimulus cycle. (C) Spectrograms for the same neuron as in (A), with corresponding power spectral densities (PSD) in the insets to the right. Note that the temporal patterning begins almost immediately and lasts for the duration of the stimulus, and that the fundamental frequency in the neural response reflects the frequency of the sinusoid. (D) Average spectrograms computed from the population of neurons that exhibited significantly phase-locked responses above 200 Hz (11 neurons in area 3b, five neurons in area 1). (E) Proportion of neurons in areas 3b and 1 that produced phase-locked responses at each frequency tested. (F) Traces of 1,000 action potential waveforms for the neuron whose responses are shown in (A–C); scale bar = 250 µs. (G) Spectrograms of neural responses to sine-sweep stimuli with either upward or downward sweeps between 50 and 400 Hz (left, same neuron as in (A); right, average over phase-locked population).

First, we found that the responses of individual neurons were often phase-locked to sinusoidal stimuli; that is, neurons tended to respond within restricted portions of the stimulus cycle (Figure 2A–2C). Surprisingly, a subpopulation of neurons exhibited phase-locked responses to sinusoids above 200 Hz (henceforth referred to as phase-locked or PL neurons), with some neurons phase-locked at frequencies up to 800 Hz (Figure 2D), implying a spiking precision of less than 1 ms. Across the population, 49% of neurons exhibited some entrainment as measured by deviation from a uniform phase-response distribution (Hodges-Ajne test with Bonferroni correction). Split by area, 86% of area 3b neurons and 43% of area 1 neurons, but only 3% of area 2 neurons showed some form of entrainment. In area 3b, the majority of neurons produced phase-locked responses at the lowest frequency used, i.e., 50 Hz, with 95% of all entrained neurons being modulated significantly at that frequency. Phase locking then decreased with increasing frequency down to 4% at 800 Hz (Figures 2E and S4). The degree of entrainment in the responses of phase-locked neurons to sinusoids was comparable to that reported in barrel cortex (vector strength, r = 0.35±0.17 and 0.18±0.09 at 50 and 800 Hz, respectively) [13].

**Figure 3:**
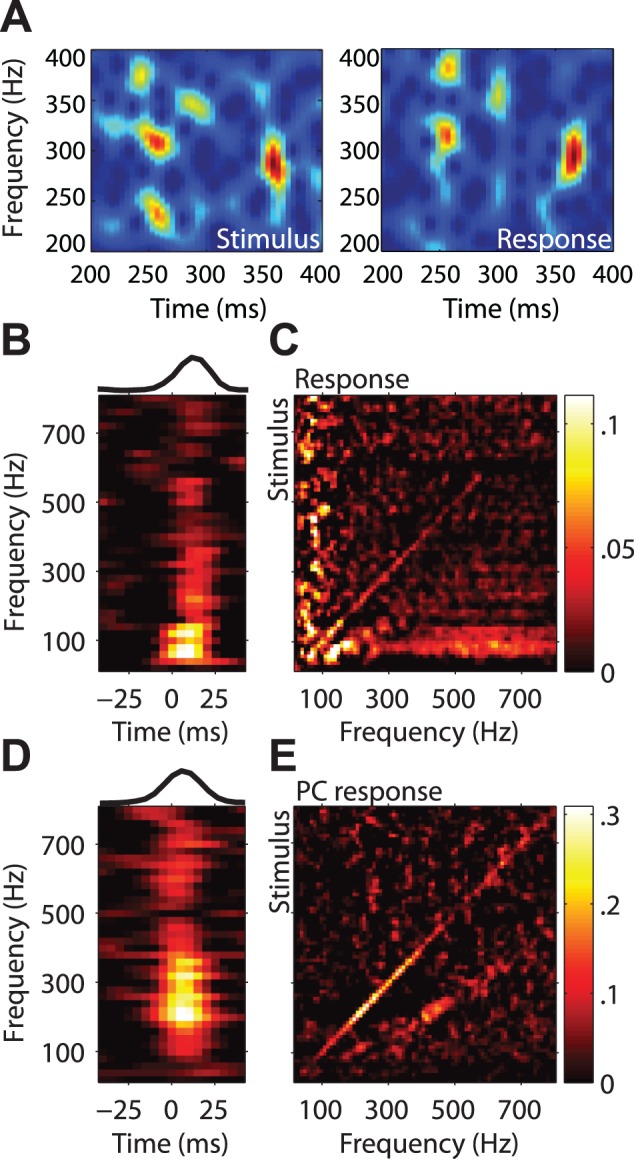
Cross-correlation of the spectral content of the stimulus with the spectral content of the response. (A) Spectrogram of one band-pass noise stimulus (left) and corresponding spectrogram of a single neuron response (right). (B) Average cross-correlation of stimulus and response spectrograms for the population of PL neurons, showing correlations between stimulus frequency and responses at the same frequency for different lags. Top inset: cross-correlation averaged across frequencies (peak lag at ∼18 ms) (C) Cross-correlations as in (B), but showing stimulus versus response frequency at the optimal lag. (D,E) Same as in (B,C) but for a population of PC afferents (peak lag at ∼5 ms).

Sinusoidal stimuli are steady-state stimuli that are rarely if ever encountered in everyday tactile experience. We therefore assessed whether the spectral content of more naturalistic stimuli was also represented in the timing of cortical responses. To this end, we computed the cross-correlation between the spectrograms of band-pass noise stimuli and the spectrograms of the evoked neuronal responses. The objective of this analysis was to ascertain whether the instantaneous frequency content of the stimulus (i.e., the frequency content during short stimulus epochs) was reflected in the instantaneous frequency content of the response. We found that neurons that exhibited entrained responses to sinusoidal stimuli also exhibited significant entrainment to noise stimuli for frequencies up to 800 Hz (see Figure 3A–3C). In fact, the degree of phase locking in cortical neurons was similar to that observed in PC afferents (Figure 3D and 3E). Entrained responses were also observed for other non-stationary stimuli, such as sinusoidal and diharmonic sweeps (Figures 2G and S5).

**Figure 4:**
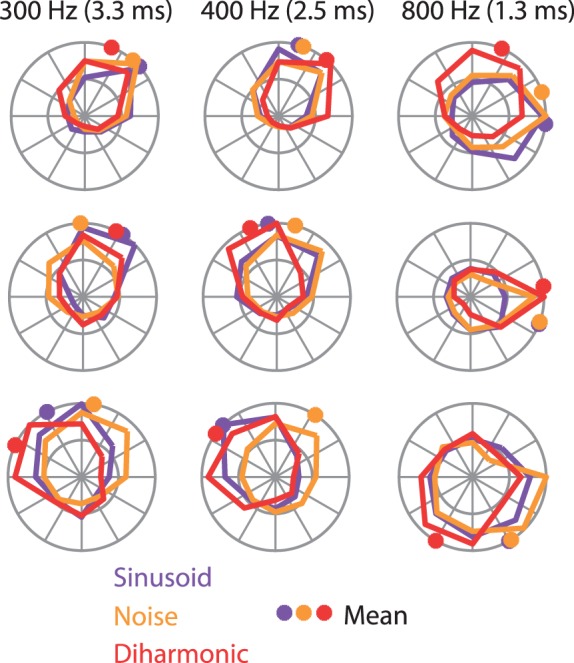
Phase tuning for complex stimuli. Normalized histograms showing phase tuning for sinusoidal (purple), diharmonic (red), and band-pass noise stimuli (orange) at three different frequencies (indicated at the top, along with the corresponding cycle duration) for three example neurons (rows). Colored dots indicate preferred phase. The phase tuning is highly consistent across stimulus types.

Coding frequency based on response entrainment requires that spikes occur within restricted phases of each stimulus cycle. That way, frequency information is preserved even if the neuron does not spike on every stimulus cycle. Furthermore, information conveyed through spike-timing can be more readily combined across neurons if the phase of the response is consistent across different stimulation conditions. With this in mind, we demonstrated that the phase of the entrained response of each neuron at each frequency was consistent across stimulus types, as evidenced by the fact that the angular deviance between the preferred phase calculated across the three different datasets was significantly smaller than would be expected by chance (27±16° compared to 50±18°, *p*<0.01, *t*(1,041) = −15.4, two-sample *t*-test) (Figure 4). In other words, when a neuron phase locked to a given frequency component, it did so at the same phase, regardless of whether that component occurred in isolation or in combination with other frequency components.

In conclusion, a subpopulation of neurons in areas 3b and 1 systematically produces phase-locked responses to skin vibrations up to 800 Hz. This precise spike timing may thus constitute the neural basis for our ability to discern the frequency content of both simple and complex skin vibrations.

### Multiplexing Stimulus Information and Neural Correlates of Perceptual Judgments

To explicitly test whether the representation of stimulus amplitude and frequency is multiplexed in S1, we quantified the amount of information conveyed in the strength and timing of the neuronal responses about these two stimulus properties (see Materials and Methods). Additionally, we sought to establish a link between neuronal responses and human perceptual judgments, as a further test of the proposed coding schemes.

**Figure 5:**
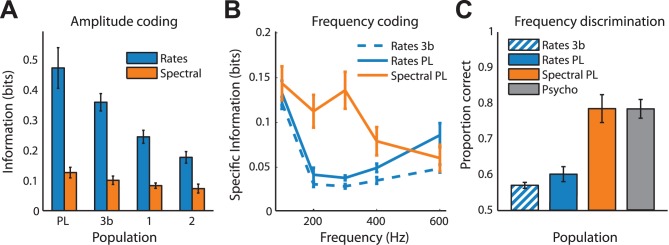
Multiplexing of stimulus information. (A) Mean mutual information between stimulus amplitude and firing rates (blue) and between stimulus amplitude and spectral peak (orange), computed from the responses of PL neurons, and neurons in areas 3b, 1, and 2. (B) Mean specific information about stimulus frequency conveyed in the response rates and timing of S1 neurons. (C) Performance on a frequency discrimination task (300 Hz versus 200 or 400 Hz) predicted from the firing rates of individual neurons in area 3b, from the rates of individual PL neurons (blue), and from the spectral peak of the responses of PL neurons (orange). Performance of human subjects is shown in grey. Error bars denote the standard error of the mean on all panels.

For sinusoids, we found that the firing rates of individual neurons conveyed significantly more information about stimulus amplitude than did the frequency content of the neural response (specifically the spectral peak), which was mostly uninformative (repeated measures ANOVA: *F*(1,146) = 173.0, *p*<0.01, Figure 5A). Stimulus frequency, on the other hand, was mainly encoded in the frequency content of the neural response (rate versus spectral peak for phase-locked neurons across all frequencies: *F*(1,75) = 17.4, *p*<0.01, Figure 5B), especially in the range from 200 to 400 Hz, which is important in texture perception [14]. Firing rates, on the other hand, are most informative about the lowest frequencies, consistent with previous findings (note that some of the increase in information in firing rates at the lowest and highest frequencies can be attributed to the differences in the amplitudes used at those frequencies, see Materials and Methods) [10],[12]. Thus, PL neurons not only conveyed information about stimulus frequency in the frequency content of their responses, but also conveyed more information about stimulus amplitude in their firing rates than did the other neurons in areas 3b and 1 (two-sample *t*-test: *t*(116) = 4.2, *p*<0.01). Thus, information about stimulus frequency and amplitude is multiplexed within a single population of neurons.

To assess how these observed differences in information about frequency might be reflected in perceptual performance, we calculated neurometric functions for a subset of the sinusoidal stimuli (see Materials and Methods). We found that, while 300 and 400 Hz are barely discriminable from 200 Hz based on firing rates, they are readily so based on the spectral content of neural responses, in agreement with human psychophysical performance (Figure 5C).

To extend our analysis to the noise data, we binned stimulus and responses into short time windows (<75 ms) and calculated the mutual information between the instantaneous rate or frequency composition of the response and the instantaneous amplitude or frequency content of the stimulus (see Materials and Methods). Results from this information-theoretic analysis were consistent with those obtained from the sinusoidal data, with firing rates conveying more information about the amplitude and frequency content of the responses conveying more information about frequency content of the stimulus (Figure S6). As it is unclear to what extent changes in frequency over short time windows are perceptually available, we could not straightforwardly isolate the contribution of the rapidly changing frequency composition of the noise stimuli to their perceptual discriminability. Instead, we had human subjects rate the overall dissimilarity of pairs of complex vibrations (band-pass noise) in order to assess the extent to which perceptual dissimilarity was correlated with differences in temporal patterning, as reflected in inter-spike interval (ISI) distributions, or whether dissimilarity could be predicted on the basis of differences in firing rate alone.

**Figure 6:**
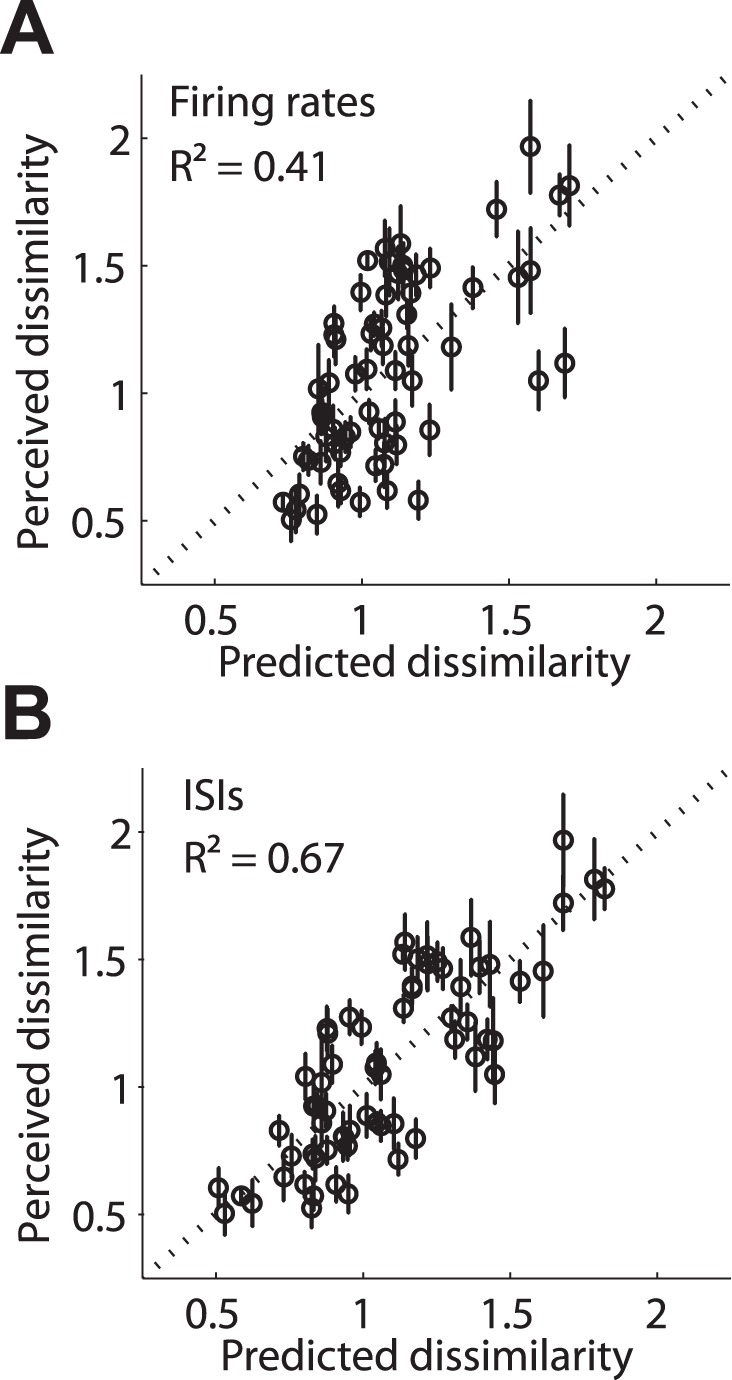
Predicting psychophysical responses to noise stimuli. Predicted versus perceived dissimilarity as calculated from (A) firing rates, and (B) ISIs. Each dot represents the mean perceived dissimilarity of one pair of stimuli. Predictions based on ISI distributions are significantly better than those based on firing rates alone, suggesting that spike timing contributes to perception. Error bars denote standard error of the mean.

Using responses of PL neurons, we found that predictions that included spike timing and firing rate (ISI histograms encompass both) outperformed predictions that were based on firing rate alone (*R^2^* = 0.67 versus 0.41; *δR^2^* F-test: *F*(65,65) = 1.75, *p*<0.05), suggesting that the temporal patterning in the response of S1 neurons shapes perception (Figure 6). Importantly, model performance is consistent with what would be expected if amplitude is encoded in the rates and frequency in spike timing: differences in (log) stimulus amplitude accounted for 27% of the variance in dissimilarity judgments while amplitude and frequency together accounted for 74% of the variance (Figure S7). We verified that the advantage of spike timing was not due to the coarse modulation of the firing rate that follows the stimulus envelope; indeed, spike trains with rate modulations matched to cortical responses but Poisson spiking yielded the same performance as firing rates (see Materials and Methods). Furthermore, spike timing outperformed firing rates when predicting ratings obtained from individual subjects (paired *t*-test on Fisher z-transformed correlations, *t*(7) = 7.09, *p*<0.001), or when using different approaches to compute spectral difference (e.g., based on power spectra of the neuronal responses, unpublished data). Our psychophysical results are consistent with the interpretation that the spike timing in areas 3b and 1 not only contains stimulus information but also shapes tactile perception, as is the case at the somatosensory periphery [7]. While predicting human perceptual judgments from macaque neuronal responses has a long, fruitful history, and humans and macaques have been shown to perform identically on vibratory discrimination tasks (see e.g., [15]–[18]), a more conclusive demonstration of the importance of spike timing in shaping perception will be to show that trial-to-trial fluctuations in phase locking are related to the animal's perception.

## Discussion

Our results support the emerging idea that different sensory attributes are encoded at different temporal resolutions in the same neural populations, leading to a multiplexed code [19],[20]. Indeed, PL neurons simultaneously convey information about both the amplitude and the frequency composition of simple and complex skin vibrations. Stimulus amplitude, which changes relatively slowly, is represented at a relatively coarse temporal resolution (tens to hundreds of milliseconds), while stimulus frequency is represented by precisely timed action potentials (on the order of one millisecond).

At the somatosensory periphery, stimulus amplitude is encoded in the strength of the responses of the three populations of cutaneous mechanoreceptive afferents [5], a rate-based representation of stimulus intensity that we show is preserved in S1. However, the rate-intensity functions of afferents are highly frequency-dependent, particularly for rapidly adapting (RA) and PC fibers. A neuron receiving primarily (unprocessed) PC or RA input would thus exhibit highly frequency-dependent responses, yet rate-intensity functions of S1 neurons are almost completely frequency-independent. How frequency-dependent afferent responses are combined and processed to yield a consistently frequency-independent representation of stimulus amplitude in S1 remains to be elucidated.

Stimulus frequency is reflected in the precise timing of the spiking responses of mechanoreceptive afferents [7],[8], a temporal representation of stimulus frequency that is preserved in areas 3b and 1. The behavioral relevance and even presence of precise spike timing in sensory cortical responses has remained controversial: some groups observe it and ascribe to it a sensory role [13],[17],[21], while others either do not observe it [22],[23] or dismiss it as an epiphenomenon [12]. Our results suggest that information about the frequency composition of skin oscillations is conveyed in the timing of cortical responses and that this timing shapes perception. Indeed, information about the frequency content of tactile vibrations is perceptually available, even at frequencies above 100 Hz, a perceptual ability that cannot be mediated by a rate code in areas 3b and 1.

A time-based code for frequency for stimuli in the so-called “vibration” range (>100 Hz) [8] stands in contrast to the proposed code for frequency in the “flutter” range (<50 Hz). Indeed, at flutter frequencies, while both the rate and timing of S1 responses convey information about stimulus frequency, firing rates are more predictive of judgments of stimulus frequency [12]. Our results suggest that individual stimulus cycles of a flutter stimulus are encoded as waxes and wanes in amplitude and constitute distinct neural events, the amplitude and frequency of which is encoded in neuronal firing rates. In contrast, the frequency composition of stimuli in the vibration range is reflected in much more temporally precise patterns of spiking. The presence of two distinct coding schemes for the same stimulus property (frequency composition) is not surprising given that stimuli in the flutter and vibration range (a) are mediated by distinct populations of mechanoreceptive afferents at the somatosensory periphery (RA versus PC fibers) (b) elicit qualitatively different perceptual experiences, and (c) likely mediate different aspects of tactile perception.

Touch is often likened to vision: In both systems, stimulus information is inferred from a spatio-temporal pattern of activation across a two-dimensional sensory sheet (the retina and the skin). Somatosensory neurons exhibit many of the same properties—e.g., center surround receptive fields (RFs) [24], orientation [25], and motion selectivity [26],[27]—as do their visual counterparts. However, natural tactile experience —e.g., texture exploration—often involves skin oscillations that spread across the skin almost unaltered [28] and convey stimulus information [14]. These skin oscillations result in high-frequency entrainment in the responses of mechanoreceptive afferents that is preserved in the responses of S1 neurons. Such high-frequency entrainment has also been observed in auditory cortex of primates [21] and in barrel cortex of rodents [13]. Our results thus suggest a complementary processing mode for the somatosensory system, one that draws analogies with the auditory system or the vibrissal system. Co-existing in S1, then, are spatial representations and temporal ones, mediated by rate-based and timing-based codes.

## Materials and Methods

### Behavioral Training

Prior to the recording sessions, all animals were trained to sit in a primate chair with their heads fixed and hands restrained, and habituated to the test apparatus. In order to maintain alertness during the recording sessions, the animals performed a simple visual discrimination task. Briefly, the animals fixated on a small square presented at the center of the field of view on an LED monitor located in front of the animal. After fixating for approximately 1 s, two circles of different luminance appeared to the left and right of the central fixation point, and the animal was given a juice reward for making a saccade to the brighter of the two. The relative luminance of the two dots was adjusted to render the task sufficiently challenging to keep the animal engaged. Eye movements were tracked using Arrington Research Eye Tracker (ViewPoint PC-60, Arrington Research) and visual stimuli presented using in-house software based on the OpenGL library.

### Surgery

First, a custom-made head-holding device was secured to the skull and allowed to osseointegrate for 1 mo before head-fixing. Once the animals were sufficiently habituated to the test apparatus and trained to perform the task, a recording chamber was attached to the skull using bone cement such that it circumscribed the hand representation in S1 (as determined using stereotaxic coordinates, AP +6, ML +22), and a craniotomy was made over the internal diameter of the chamber. Surgical anesthesia was induced with ketamine HCl (20 mg/kg, IM) and maintained with Isoflurane (10–25 mg/kg/h). All surgical procedures were performed under sterile conditions and in accordance with the rules and regulations of the University of Chicago Animal Care and Use Committee.

### Neurophysiological Procedures

#### Cortical experiments

Extracellular recordings were made in the postcentral gyri in four hemispheres of two macaque monkeys using previously described techniques [25]–[27]. On each recording day, a multielectrode microdrive (NAN Instruments) was loaded with two to four tungsten electrodes (FHC Inc.). These electrodes are initially fully insulated but a small area of the tip becomes dis-insulated upon penetration through the dura. Tip resistances following dural penetration typically range from 100–200 kΩ. The microdrive guide tubes were lowered into a recording chamber filled with 3% agar and oriented so that the electrodes were normal to the cortical surface. The electrodes were then driven into the cortex until they encountered neurons in area 1 with RFs on the distal fingerpad. A day spent recording from area 1 was typically followed by a day spent recording from area 3b or area 2. When recording from area 3b, the electrodes were driven 2–3 mm below the top of the neural activity until neurons with RFs on the distal fingerpad were encountered. The transition from area 1 to area 3b exhibits a characteristic progression of RF locations. As one descends from the cortical surface through area 1 into area 3b, the RFs progress from the distal, to middle, to proximal finger pads, and then to the palmar whorls. Within area 3b, the RFs proceed back up the finger, transitioning from proximal, to medial, and ultimately to distal pads. The representation of the digits in area 2 lies just caudal to, and mirrors that of area 1. Thus, when moving the electrode caudally from area 1 to area 2 one first encounters the representation of the distal pads, and then more caudally, the medial and proximal pads. The most salient functional feature identifying area 2 is the presence of neurons with response properties that are either purely proprioceptive, or that contain both cutaneous and proprioceptive properties. Because of the apposition of the distal finger pad representation in areas 1 and 2, we used the presence of these proprioceptive neurons to inform our categorization. We recorded from neurons whose RFs were located on the distal pads of digits 2–5. On every second day of recording, the electrode array was shifted 200 µm along the postcentral gyrus until the entire representation of digits 2–5 had been covered. At the end of the recording day, the electrodes were withdrawn and a few drops of dexamethasone were applied to the dura. The chamber was filled with sterile saline and sealed. Recordings were obtained from neurons in areas 3b, 1, and 2 that met the following criteria: (1) action potentials were well isolated from the background noise, (2) the RF of the neuron was on the glabrous skin, and (3) the neuron was clearly driven by light cutaneous stimulation.

#### Peripheral experiments

Experimental procedures have been previously described [5] and are only summarized here. All experimental protocols complied with the guidelines of the Johns Hopkins University Animal Care and Use Committee and the National Institutes of Health Guide for the Care and Use of Laboratory Animals. Single unit recordings were made from the ulnar and median nerves of two anesthetized Macaque monkeys (*M. mulatta*) using standard methods [8]. Standard procedures were used to classify mechanoreceptive afferents according to their responses to step indentations and vibratory stimulation [8],[9]. An afferent was classified as PC if (1) it was vigorously activated by air blown gently over the hand; (2) it was activated by transmitted vibrations produced by tapping on the hand restraint; and (3) its RF was large. The point of maximum sensitivity of the afferent (or hotspot) was located on the skin using a handheld probe and then marked with a felt-point pen. The stimulator probe was centered on the hotspot of the afferent to the extent possible (PC RFs do not have clear hotspots). The tip of the probe was fixed with cyanoacrylate glue to the skin at its resting position, i.e., with no pre-indentation.

### Stimuli

Tactile stimuli were delivered to the distal pads of the digits using a stainless steel probe with a 2 mm tip diameter driven by a shaker motor (LW–132–7 Electrodynamic Shaker System, Labworks Inc.). The shaker motor was calibrated before each experimental run such that stimuli were highly accurate and repeatable (see [5] for details about the calibration procedure). The stimulus traces were reconstructed from the acceleration output of the accelerometer (8702B50M1, Kistler Instruments Corp.) by numerical integration and high-pass filtering (using a 25-Hz cut-off frequency). We verified the accuracy of the reconstructed traces by comparing them to the idealized traces (i.e., the motor commands) and to measurements obtained using a Laser Doppler vibrometer (Polytec OFV-3001 with OFV 311 sensor head, Polytec, Inc.) [28]. We delivered three types of stimuli that varied in spectral complexity, including simple sinusoids, diharmonic stimuli, and band-pass noise (cf. [5]). The ranges of frequencies and amplitudes spanned or exceeded the ranges experienced in everyday tactile experience [14]. 

#### Sinusoids

Sinusoidal stimuli were delivered at 50, 100, 200, 300, 400, 600, and 800 Hz. Amplitude ranges were 34–680 µm, zero to peak, at 50 Hz, 15–300 µm at 100 Hz, 7–133 µm at 200 Hz, 4–82 µm at 300 Hz, 3–59 µm at 400 Hz, 4–36 µm at 600 Hz, and 3–26 µm at 800 Hz. At each frequency, amplitudes were incremented in 10 equal logarithmic steps over the range.

#### Diharmonic stimuli

Diharmonic stimuli were created by the addition of two sinusoids using the following equation:
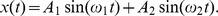
where *A*_1_ and *A*_2_ are the amplitudes of the low and high-frequency components, respectively, and ω_1_ and ω_2_ are the two frequencies (ω_1_<ω_2_). Component frequencies ranged from 50 to 800 Hz, component amplitudes from 1 to 600 µm, and depended on stimulus frequency (see frequency dependence of ranges for sinusoids above). We presented three sets of diharmonic stimuli, where the component amplitudes were scaled such that they were equal in peak position (indentation depth), velocity, or acceleration.

#### Band-pass noise

Each noise stimulus was created from the same wide-band noise trace and filtered to the specified frequency range. A total of 16 frequency ranges were included (Table 1). Just as for diharmonic stimuli, there were three subsets of noise stimuli, whose frequency spectra were flat in position, velocity, or acceleration (noise_P_, noise_V_, or noise_A_). Each noise stimulus was then scaled to one of four root-mean-square (*rms*) amplitudes that spanned one order of magnitude; *rms* amplitudes depended on the band-pass and ranged from 0.5 to 300 µm.

**Table 1:** Low- and high-frequency cut-offs for noise stimuli.

*ω_1_ (Hz)*	*ω_2_ (Hz)*
50	100, 200, 300, 400, 600, 800
100	200, 300, 400, 600, 800
200	400, 600, 800
300	600
400	800

### Psychophysical Procedures

All testing procedures were performed in compliance with the policies and procedures of the Institutional Review Board for Human Use of the University of Chicago. All subjects were paid for their participation, and reported normal tactile function and no history of neurological disease. The stimulator was encased in a sound attenuating chamber (cf. [29]) and subjects wore earbuds playing pink noise inside of sound attenuating earphones to eliminate auditory cues. Before each experimental block, the stimulus probe was lowered until it just contacted the skin. Each trial was preceded and followed by a 1 s period of no stimulation to reduce the effects of vibratory adaptation [30],[31]. On each trial, two vibratory stimuli were presented, for one second each, with 0.2 s between the stimuli.

#### Frequency discrimination

Seven subjects (2 females, 5 males), ranging from 21 to 31 y of age, participated in this experiment. Subjects were asked to indicate whether the second of two consecutively presented stimuli was higher or lower in frequency than the first. Subjects received feedback after each trial about whether their decision was correct or not. One of the stimuli, the standard, was always a 300-Hz sinusoid, while the other, the comparison, was either a 200- or 400-Hz sinusoid, with the order chosen pseudo-randomly. Each stimulus was presented at one of five amplitudes, chosen pseudo-randomly from a subset of amplitudes used in the neurophysiological experiments (at that frequency). Amplitudes ranged from 9 to 35 µm at 200 Hz, and from 11 to 42 µm at 300 and 400 Hz; each standard was paired with each comparison once per experimental block yielding 50 trials (two comparison frequencies×25 possible amplitude pairs). Each subject participated in three experimental blocks; the first block was for practice and was discarded from later analysis.

#### Noise dissimilarity ratings

Eight subjects (5 females, 3 males), ranging from 19 to 35 years of age, participated in this experiment. Subjects were asked to estimate the perceived dissimilarity of two consecutively presented band-pass noise stimuli on a ratio scale: If the stimuli felt identical, the subject was to assign the number 0 to the pair. In a given experimental block, the first rating was arbitrary (unless the stimuli felt identical). The subjects were instructed to produce a number twice as large if the next stimulus pair felt twice as dissimilar, or, a number one-half as large if it felt one-half as dissimilar. They were encouraged to use decimals or fractions as deemed necessary. Before the first experimental block, subjects rated a subset of pairs for practice. The practice block was followed by five experimental blocks in which archival data were collected. Psychophysical ratings were normalized by the mean rating obtained in each experimental block, then averaged across blocks and subjects. The stimuli presented were a subset of the band-pass noise stimuli used in the electrophysiological part of the study, namely 50–100, 50–200, 200–400, and 400–800 Hz from the noise_A_ set; 50–200, 100–300, 100–600, and 200–800 Hz from the noise_V_ set; and 50–200, 100–200, 200–400, and 200–800 Hz from the noise_P_ set. Amplitudes of the different stimuli were equated to the extent possible in order to reduce the influence of differences in stimulus amplitude on the subjects' perceived dissimilarities.

### Analysis

All analyses were performed using Matlab (The Mathworks). Circular statistics were computed using the CircStat toolbox [32].

#### Frequency dependence of firing rates

The objective of this analysis was to determine whether neuronal firing rates were significantly modulated by stimulus frequency. To this end, we fit the relationship between firing rates and stimulus amplitude to functions of the form:
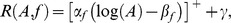
where *R(A,f)* is the mean firing rate evoked by a stimulus at frequency *f* and amplitude *A*, []^+^ denotes rectification, and *α_f_* and *β_f_* are the slope and threshold and γ is the baseline firing rate (*α_f_*, *β_f_*, and γ are free parameters). The frequency-independent model comprised only one slope and one threshold parameter, so the firing rate was assumed to be independent of the stimulus frequency. The frequency-dependent model comprised one slope and one threshold per frequency condition (seven frequencies for sinusoids, yielding 15 parameters, 16 band-passes for noise, yielding 33 parameters). We then compared statistically the ability of the two models to account for the neuronal firing rates by fitting each model to the responses to four of the five repeated presentations of each stimulus and testing the model's predictions on the fifth response. Specifically, we assessed whether the frequency-dependent model yielded significantly better predictions than did the frequency-independent model as assessed by a δ*R^2^* F-test. As described in the Results, when we ran these models, we found that the frequency-dependent model yielded better predictions than the frequency-independent model for only a very small proportion of neurons. That proportion became essentially nil when we eliminated frequency conditions that included 50 Hz. In other words, rate-intensity functions are highly frequency independent at 100 Hz and above.

#### Determination of phase angle and entrainment

To determine the phase of each spike with respect to the stimulus, we first calculated the lag between the stimulus and the neural response by cross-correlating the reconstructed stimulus traces with a low-pass filtered version of the neural response for each neuron. Lags were 21±7.5 ms on average, which is well within the range that would be expected for neurons in S1. The stimulus trace was then offset by each individual neuron's lag for subsequent calculations. In order to determine a unique stimulus frequency at the time of each spike, we defined a stimulus cycle as the length of time between two consecutive upwards zero-crossings of the stimulus trace. Using spectrograms calculated from the same stimulus traces, we verified that our method correctly tracked the dominant frequency over time. Using alternative criteria such as the length of time between consecutive peaks of the stimulus traces lead to almost identical results. Given this measure of cycle length, we could then calculate the phase of each individual spike with respect to the current stimulus cycle. To compare the consistency of phase tuning across the different stimulus types (sinusoids, diharmonic, and noise), we calculated the preferred phase for each neuron at different frequencies for each stimulus type and then computed the angular deviation between those phases. The resulting angular deviations were then compared to those calculated from neurons that did not exhibit significant phase locking (and whose preferred phase should therefore vary randomly).

#### Cross-correlations of power spectra

To test whether the spectral content of the neural responses mirrored the spectral content of the band-pass noise stimuli, we computed spectrograms of all band-pass noise position traces and the corresponding spike trains for individual neurons. Because of the trade-off between time and frequency resolution inherent to spectrograms, we used two different parameter sets: For a higher temporal resolution, we used 50-ms windows, resulting in a frequency resolution of 20 Hz. For a higher spectral resolution, we used 75-ms windows, with a frequency resolution of 13.3 Hz. The step size was 5 ms in either case. We then cross-correlated the resulting stimulus and response spectrograms across all frequency pairs. Thus, we examined how well the presence of a particular stimulus frequency correlated with the presence of a particular response frequency. In order to remove artifacts in the power spectra due to coarse modulation of firing rates by the stimulus envelope, we subtracted spectral cross-correlations calculated from rate-modulated Poisson spike trains (see below) from our original estimates. We verified that this procedure did not qualitatively change our results.

#### Generating rate-modulated Poisson spike trains

First, recorded spike trains were convolved with a Hann window of 50 ms total length. Next, action potentials were sampled from this inhomogeneous rate. The resulting spike trains show virtually identical rates and coarse time-varying rate modulations as the original data, but precise spike timing is abolished.

#### Information theoretic analysis

To calculate the mutual information between neuronal responses and stimulus frequency for the sinusoidal data, we pooled trials from all amplitudes (ten per frequency) and repetitions (five per amplitude-frequency pair), which resulted in 50 individual trials per frequency for each neuron. Similarly, when calculating the mutual information between neuronal responses and stimulus amplitude, we ordered the stimuli by amplitude, and split them into bins such that each bin included 50 stimuli. To reduce the dependence of amplitude and frequency in our stimulus set, we eliminated from our analysis data from trials with 50- or 800-Hz components, at which amplitudes were completely non-overlapping (see Figure S8 for the remaining dependence). To characterize the response entropy based on firing rates, we counted the number of spikes within the full 1-s window and split these spike counts into seven bins of equal width. To characterize the response entropy based on spike timing, we computed the peak frequency from the power-spectral density of the response spike trains and split these into seven bins. To perform an analogous analysis on the noise data, we used the Hilbert transform of the stimulus traces (filtered to below 25 Hz) as our measure of instantaneous stimulus amplitude, and the spectral centroid of the stimulus spectrograms as our measure of the frequency, and sampled these in 5-ms steps (see below for description of the sampling process). We restricted our analysis to time windows in which the stimulus amplitude was greater than the grand mean of the stimulus amplitude to remove episodes with low spiking and ensure clearly defined peaks in the stimulus spectrograms (in addition, the frequency composition of peri-liminal stimuli is not perceptually available, see [8]). Instantaneous amplitudes ranged from 30 to 750 µm and were placed into 20 bins, such that each bin contained the same number of trials. Spectral centroids ranged from 65 to 300 Hz and were also divided into 20 bins with equal number of observations. Single-trial time-varying firing rates were extracted by counting spikes within 55-ms wide bins and correcting for response latency. To compute the frequency composition of the response, we again took the peak frequency of single-trial response spectrograms (calculated as described above). As spike counts ranged from 0 to 21 spikes, we restricted the time-based code to 22 response bins to ensure a fair comparison. Our stimulus selection resulted in more than 2,200 samples for each amplitude or frequency condition (i.e., 44,000 samples in total). Mutual information was calculated as:
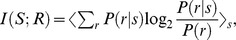
where *P(r|s)* denotes the probability of observing a response *r* given stimulus *s*, *P(r)* denotes the marginal probability of response *r*, and angle brackets denote the average over all stimuli *s* (before averaging, the quantity *I(s;R)* denotes specific information [33], shown in Figure 5B). To correct for any bias introduced by under-sampled data, we calculated a bias term for each neuron based on the Panzeri-Treves method [34] and subtracted this term from our estimated mutual information values. For a good estimate of the bias, the number of trials for each condition should be larger than the number of possible responses, a condition that was fulfilled. We found that the bias was small compared to the effect sizes (<10%) for the sinusoidal data and negligible for the noise data. Neurometric functions were computed from the mutual information estimates for a subset of the sinusoidal data using Fano's inequality [35].

#### Predicting perceived dissimilarity from neural responses

We compared perceived dissimilarities with differences in the neural responses of the same stimulus pairs. In order to examine the contributions of rate-based and spike-timing-based codes on the perceived dissimilarities, we calculated two different neural dissimilarity measures: (1) a rate-based measure where the dissimilarity between two conditions is calculated as the average absolute difference in spike counts across a neural population; (2) a spike timing-based measure where the dissimilarity is calculated from average absolute differences in ISI histograms. ISI histograms were restricted to intervals between 2 ms and 50 ms (containing the vast majority of ISIs), and were divided into eight bins of equal width. We tried alternative methods for determining neural dissimilarities based on spike timing, namely differences in the power spectra of evoked responses, and a spike distance measure that calculates the cost of transforming one spike train into another at a given temporal resolution [36], and found qualitatively comparable results in that these measures outperformed dissimilarities based on differences in rate alone.

## Supporting Information

Figure S1 **Rate-intensity functions.** (A) Rate-intensity function for an individual neuron computed from sinusoidal stimuli. Colors correspond to different frequencies, the dark trace shows the rectified logarithmic function fit to this neuron (shown at the top). Error bars show standard error of the mean. As was typically the case, the firing rate of this neuron is not significantly modulated by the stimulus frequency. (B) Rate-intensity function for the same neuron as in (A), computed from band-pass noise stimuli. Colors change from dark to bright red with increasing average stimulation frequency. Markers correspond to different noise types (triangle, noise_A_; circle, noise_V_; square, noise_P_, see Materials and Methods for explanation). (C) Population rate-intensity functions for areas 1 and 2, computed from sinusoids. (D) Same as in (C), but computed from band-pass noise stimuli.
(EPS)

Figure S2 **Amplitude coding for a band-pass noise stimulus.** The black trace shows the time-varying stimulus, the grey trace, the envelope (computed using the Hilbert transform). The response of neurons in areas 3b and 1 tracks the waxing and waning of the stimulus envelope. The amplitude signal is weaker in area 2.
(EPS)

Figure S3 **Frequency-independence of firing rates.** (A) Mean firing rate of neurons in area 3b as a function of frequency, at three different amplitudes (5, 10, and 25 µm). Firing rates were computed from rate-intensity functions (see Materials and Methods). We do not show the responses at 50 and 100 Hz because the minimum amplitudes at those frequencies were higher than 10 µm and we wished to avoid extrapolating. (B) Histogram of frequency dependent firing rate slopes at 25 µm for all 3b neurons. The firing rates of individual neurons are only marginally dependent on frequency.
(EPS)

Figure S4 **Frequency ranges for all entrained neurons.** Bars indicate the range over which individual neurons exhibit significant phase locking to sinusoidal stimuli. Inset: Action potential waveforms (1,000 traces each) for four exemplary neurons that entrain over different frequency ranges (NPL indicates a neuron that did not phase lock at any frequency). That individual neurons (1) can produce entrained responses over a wide range of stimulus frequencies (50–800 Hz) and (2) tend to peak in entrainment at 50 Hz, suggests that individual neurons receive convergent input from multiple cutaneous submodalities (cf. [37]). Indeed, the frequency range of entrainment implicates PC afferents while the peak at low frequencies implicates other (most likely RA) afferents.
(EPS)

Figure S5 **Neural responses to diharmonic sweeps.** (A) Spectrograms of two diharmonic sweep stimuli. (B) Spectrogram of the response elicited in an individual neuron.
(EPS)

Figure S6 **Multiplexing of stimulus information for noise stimuli.** (A) Mean mutual information between instantaneous amplitude and firing rates (blue) or spectral peak (orange), computed from the responses of PL neurons, and neurons in areas 3b, 1, and 2. (B) Mean mutual information between spectral content and firing rates (blue) or spectral peak (orange), computed from the responses of PL neurons, and neurons in areas 3b, 1, and 2. Mirroring the results with sinusoids, mutual information between amplitude and firing rates was higher than between amplitude and spectral composition (*F*(1,146) = 34.7, *p*<0.01); mutual information between peak frequency of the stimulus and peak frequency of the response was higher than between peak frequency of the stimulus and response rate (*F*(1,146) = 33.1, *p*<0.01). While information about the instantaneous stimulus amplitude is low in the spectral response across all populations, PL and area 3b neurons convey information in their time-varying firing rates. Conversely, information about time-varying frequency composition is mostly conveyed in the spectral responses of neurons. Note some information about frequency in firing rates stems from the fact that the noise stimuli were biased towards lower frequencies (where firing rates do convey information) and that frequency and amplitude are not independent in our noise data on short time-scales.
(EPS)

Figure S7 **Predicting psychophysical responses from stimulus features.** Predicted versus perceived dissimilarity as calculated from (A) (log) stimulus amplitude, and (B) (log) stimulus amplitude *and* spectral centroid (average frequency, weighted by power). Symbols as in Figure 6.
(EPS)

Figure S8 **Specific information about each frequency computed from amplitude for sinusoids.** The specific information about frequency in the amplitude reflects the fact that different amplitudes were used at different frequencies. If the rates encode amplitude (and not frequency), this is the shape of the function we would expect in Figure 5B, which bolsters the argument that rates do not encode frequency.
(EPS)

## Abbreviations

S1: primary somatosensory cortex
ISI: inter-spike interval
(N)PL: (non-)phase-locked
RA: rapidly adapting
PC: Pacinian
RF: receptive field
